# Allelic and genotypic associations of *DRD2 Taq*I A polymorphism with heroin dependence in Spanish subjects: a case control study

**DOI:** 10.1186/1744-9081-3-25

**Published:** 2007-06-01

**Authors:** Jose Perez de los Cobos, Montserrat Baiget, Joan Trujols, Nuria Sinol, Victor Volpini, Enrique Banuls, Francesc Calafell, Elena Luquero, Elisabeth del Rio, Enric Alvarez

**Affiliations:** 1 Addictive Behaviours Unit of Psychiatry Department, Hospital de la Santa Creu i Sant Pau, Barcelona, Spain; 2 Genetics Department, Hospital de la Santa Creu i Sant Pau, CIBERER, Barcelona, Spain; 3 Center for Molecular Genetic – Diagnosis of Hereditary Diseases, Cancer Research Institute (IRO)-IDIBELL, Barcelona, Spain; 4 Unitat de Biologia Evolutiva, Departament de Ciències Experimentals i de la Salut, Universitat Pompeu Fabra, Catalonia, Barcelona, Spain; 5 Psychiatry Department, Hospital de la Santa Creu i Sant Pau, Avda Sant Antoni M. Claret, 167. 08025, Barcelona, Spain

## Abstract

**Background:**

Conflicting associations with heroin dependence have been found involving the A1 allele of dopamine D2 receptor gene (*DRD2*) *Taq*I A polymorphism.

**Methods:**

We compared two samples of unrelated Spanish individuals, all of European origin: 281 methadone-maintained heroin-dependent patients (207 males and 74 females) who frequently used non-opioid substances, and 145 control subjects (98 males and 47 females).

**Results:**

The A1-A1 genotype was detected in 7.1% of patients and 1.4% of controls (*P *= 0.011, odds ratio = 5.48, 95% CI 1.26–23.78). Although the A1 allele was not associated with heroin dependence in the entire sample, the frequency of A1 allele was higher in male patients than in male controls (24.4% vs. 16.3%, *P *= 0.024, odds ratio = 1.65, 95% CI 1.07–2.57). A logistic regression analysis showed an interaction between *DRD2 *alleles and gender (odds ratio = 1.77, 95% CI 1.15–2.70).

**Conclusion:**

Our results indicate that, in Spanish individuals, genotypes of the *DRD2 Taq*I A polymorphism contribute to variations in the risk of heroin dependence, while single alleles contribute only in males.

## Background

A better understanding of the etiology of heroin dependence is crucial for improving the prevention and treatment of this severe mental disorder. Genes that could be risk factors for heroin dependence have not been consistently identified; however, genetic epidemiology studies have shown that they do have an impact. These studies, with one exception [1], also suggest that such genetic factors are mainly nonspecific, because they also confer vulnerability to other substance use disorders (SUD) [2-4].

The gene coding for the dopamine receptor D2 (*DRD2*) could be involved in heroin dependence and other SUD as a nonspecific genetic factor, because opioids and other substances of abuse induce some of their rewarding effects through the mesolimbic dopamine system [5,6]. Preclinical research supports this hypothesis. An absence of opioid-rewarding effects has been reported in mice lacking *DRD2 *[7].

The *DRD2 Taq*I A is a SNP with two variants: A1, the less frequent allele, and A2. The A1 allele is associated with a reduction in the density of D2 receptors at the striatum [8-10], although one study failed to replicate this finding [11]. The hypofunction of D2 receptors associated with A1 allele could be a risk factor for SUD [12]. Healthy men who feel psychostimulant effects to be pleasant have lower D2 receptor availability than those who feel them to be unpleasant [13]. This result supports a model that links the A1 allele with SUD through the following sequence of facts: low D2 receptor density, moderate substance stimulation of dopamine system, and rewarding feelings after substance use. The addictive process could get started and continue more easily in men than in women, because D2 receptor density is lower in healthy men [9,14] as well as in heroin-dependent men [15].

A series of meta-analyses performed on 55 studies has shown an association between the *DRD2 *A1 allele and SUD in Caucasian and non-Caucasian groups [16]. However, in the particular case of opioid use disorders, associations with the A1 allele have been found in some studies [17,18], but not in others [19,20]. The aim of the present study was to examine the association of heroin dependence with *DRD2 *A1 alleles in Spanish subjects of European origin.

## Methods

### Subjects

All subjects included in this study were unrelated and their four grandparents were Spanish, of European, non-Roma origin. Patients were in methadone maintenance for treating heroin dependence (DSM-IV: 304.02;[21]), and all of them reported heroin as their primary drug of choice. Non-opioid substance use was not an exclusion criterion in the present study for two reasons: first, because heroin-dependent Spanish patients frequently report use of alcohol, benzodiazepines, cannabis, and cocaine as a secondary drug [22]; second, because *DRD2 *is potentially a nonspecific genetic factor for substance abuse or dependence. Therefore, the exclusion of heroin-dependent patients who frequently use non-opioid substances would have reduced the external validity of our study, without improving its internal validity. In the present study, non-opioid substance use was defined as a regular and/or maladaptive use pattern during a lifelong cumulative period longer than a year. Control individuals were blood donors and clinical staff members, who declared that they did not suffer SUD, with the exception of nicotine dependence. The present study was approved by the IRB of Sant Pau Hospital (Barcelona), and written informed consent was obtained from all participants.

### Genotyping

A 10-ml sample of venous blood was drawn from each participant, and DNA was isolated with a salting-out procedure [23]. All allele types were determined independently by two laboratory technicians blind to subject identity. Genotyping of *DRD2 Taq*I A polymorphism was performed by PCR as described in [24]. PCR reactions contained 10 mM Tris-HCl (pH 9.0), 50 mM KCl, 200 μM each dNTP, 1.5 mM MgCl_2_, 0.2 μM of each primer and 1.25 units of Taq Polymerase (Promega) in a 25 μl volume containing 50 ng sample DNA. The reaction mixture was denatured 4 min at 94°C, followed by 35 cycles of 94°C for 30 sec, 58°C for 30 sec, 72°C for 30 sec, and a final extension of 5 min at 72°C. Subsequently, 10 μl of the PCR product were digested with 5 units of *Taq*I restriction enzyme at 65°C overnight. The resulting products were analysed by electrophoresis and visualised under ultraviolet light. The A1-A2 genotype is revealed by three fragments (310-bp, 180-bp and 130-bp), the A2-A2 genotype by two fragments (180-bp and 130-bp), and the A1/A1 genotype is shown by an uncleaved 310-bp fragment.

### Statistical analysis

Hardy-Weinberg (HW) equilibrium was tested for the *DRD2 *polymorphism. Genotype distributions and allele frequencies were compared using χ^2^-test. Moreover, two logistic regression analyses were performed in order to examine possible interactive effects of the *DRD2 *alleles and genotypes with gender. All statistical tests were two-sided, and were considered significant at *P *< 0.05.

## Results

The group of patients comprised 281 methadone-maintained heroin-dependent individuals (Table 1). Males accounted for 73.7% of this sample. The mean (*SD*) age of the patients was 36.4 (6.3) years. Most patients reported intravenous (IV) heroin self-administration and non-opioid substance use (Table 1). In regard to these variables, patients were comparable by gender with the only exception of cannabis use: male patients reported regular and/or maladaptive patterns of cannabis use more frequently than female patients. The control group comprised 145 subjects recruited from the general population (128 blood donors and 17 clinical staff). Males accounted for 67.6% (n = 98) of this sample. The mean (*SD*) age of the control subjects was 35.3 (11.2) years. There were no statistically significant differences with regard to age or gender between the patients and the controls.

**Table 1:** Features of heroin-dependent patients^a^

	All	Males	Females	Gender comparisons	
	(*n *= 281)	(*n *= 207)	(*n *= 74)	*t *or χ^2^	P

Age (years)	36.4 ± 6.3	36.6 ± 6.2	35.8 ± 6.5	*t *= 0.92	0.36
Heroin use					
Age of onset (years)	20.1 ± 5.3	20.0 ± 5.1	20.3 ± 5.8	*t *= -0.45	0.66
Route of administration (%)					
Intravenous	70.4	73.5	62.5	χ^2 ^= 2.38	0.31
Smoked	10.8	9.5	14.3		
Intranasal	18.7	17.0	23.2		
Non-opioid substance use^b ^(%)					
Alcohol	56.6	59.9	47.3	χ^2 ^= 3.53	0.06
Benzodiazepines	42.7	43.0	41.9	χ^2 ^= 0.03	0.87
Cannabis	81.5	85.0	71.6	χ^2 ^= 6.49	0.01
Cocaine	71.5	70.5	74.3	χ^2 ^= 0.39	0.54

^a ^Values are mean ± SD unless otherwise indicated

^b ^Non-opioid substance use is defined as a regular and/or maladaptative pattern of non-opioid substance use during a longlife cumulative period longer than a year

Allele and genotype frequencies of *DRD2 *polymorphism are shown in Tables 2 and 3, respectively. Genotype frequencies were in Hardy-Weinberg equilibrium in the control group (χ^2 ^= 2.01, df = 1, *P = *0.16) but not in the patient group (χ^2 ^= 5.20, df = 1, *P *= 0.022), probably because, as described below, the latter group was enriched in A1-A1 homozygotes. A1 allele frequencies were not statistically significantly different between patients and controls (χ^2 ^= 2.18, df = 1, *P *= 0.140). Both groups differed in genotype frequency (χ^2 ^= 6.48, df = 2, *P *= 0.039). Notably, the A1-A1 genotype was detected in 7.1% of patients and 1.4% of controls (χ^2 ^= 6.43, df = 1, *P *= 0.011, odds ratio = 5.48 [95% CI 1.26–23.78]).

**Table 2:** Distribution of alleles of *DRD2 Taq*I A polymorphism in heroin-dependent patients and control individuals^a^

	All^b^			Males^c^			Females^d^		
			
Alleles	Patients	Controls	Total	Patients	Controls	Total	Patients	Controls	Total
A1	123(0.219)	51(0.176)	174(0.204)	101(0.244)	32(0.163)	133(0.218)	22(0.149)	19(0.202)	41(0.169)
A2	439(0.781)	239(0.824)	678(0.796)	313(0.756)	164(0.837)	477(0.782)	126(0.851)	75(0.798)	201(0.831)
Total	562	290	852	414	196	610	148	94	242

^a ^Normalised frequencies are given in brackets

^b ^χ^2 ^= 2.18, df = 1, *P *= 0.140

^c ^χ^2 ^= 5.08, df = 1, *P *= 0.024

^d ^χ^2 ^= 1.17, df = 1, *P *= 0.280

**Table 3:** Distribution of genotypes of *DRD2 Taq*I *A *polymorphism in heroin-dependent patients and control individuals^a^

	All^b^			Males^c^			Females^d^		
			
Genotypes	Patients	Controls	Total	Patients	Controls	Total	Patients	Controls	Total
A1-A1	20(0.071)	2(0.014)	22(0.052)	17(0.082)	0	17(0.056)	3(0.041)	2(0.043)	5(0.041)
A1-A2	83(0.295)	47(0.324)	130(0.305)	67(0.324)	32(0.327)	99(0.325)	16(0.216)	15(0.319)	31(0.256)
A2-A2	178(0.633)	96(0.662)	274(0.643)	123(0.594)	66(0.673)	189(0.620)	55(0.743)	30(0.638)	85(0.702)
Total	281	145	426	207	98	305	74	47	121

^a ^Normalised frequencies are given in brackets

^b ^χ^2 ^= 6.48, df = 2, *P *= 0.039

^c ^χ^2 ^= 8.72, df = 2, *P *= 0.013

^d ^χ^2 ^= 1.64, df = 2, *P *= 0.440

Allelic and genotypic comparisons between genders showed significant differences only in patients. In this group, the frequency of the A1 allele (Table 2) was higher in males than in females (24.4% vs. 14.9%; χ^2 ^= 5.79, df = 1, *P *= 0.016). However, differences between male and female patients regarding genotype distribution (Table 3) did not reach statistical significance (χ^2 ^= 5.37, df = 2, *P *= 0.068). DRD2 allele and genotype frequencies, however, were not significantly different between male and female controls.

Allelic and genotypic comparisons within gender showed statistically significant differences only in males (Tables 2 and 3). Unlike the results obtained with the whole sample, patient and control males differed in the frequency of the A1 allele (χ^2 ^= 5.08, df = 1, *P *= 0.024, odds ratio = 1.65 [95% CI 1.07–2.57]). The logistic regression analysis revealed an interaction between *DRD2 *alleles and gender (Table 4), such that the frequency of A1 allele was higher in patient than control males (Table 2). Similarly to the results obtained with the whole sample, patient and control males differed in genotype distribution (χ^2 ^= 8.72, df = 2, *P *= 0.013). In this case, the logistic regression analysis did not reveal an interactive effect of *DRD2 *genotypes and sex (data not shown).

**Table 4:** Logistic regression terms for DRD2 alleles and sex

Variable	Coeff	Std Err	Odds ratio	95% conf. int.
*DRD2 *× Sex	0.57	0.22	1.77	1.15–2.70
Constant	0.58	0.08	1.79	

## Discussion

The present case-control study shows that *DRD2 *contributes to the variation in the risk for heroin dependence in Spanish individuals. The A1-A1 genotype was significantly associated with heroin dependence in the entire sample, while the A1 allele was associated only in males. Accordingly, the conclusion of a series of meta-analyses is that *DRD2 Taq*I A polymorphism is associated with SUD, particularly with severe SUD [16]. We consider that the patients in the present study suffered a severe SUD, because they had a long history (≈10 years) of mostly IV heroin use, which was frequently combined with non-opioid substance use.

Regarding specific studies on opioid use disorders, our findings agree with two previous reports from Australia [17] and Iran [18], and disagree with two other reports from China [19,20] and Germany [20]. In the Australian study, patients were in methadone treatment and often used non-opioid substances, as in the present study. Both features could maximise a study's ability to detect genetic associations: with non-opioid substance use, because it is also related with the *DRD2 *A1 allele [16]; and with methadone treatment, because it can be a marker of heroin dependence severity [25].

Patients who participated in the studies showing negative results [19,20] were not in methadone treatment and, in the case of the Chinese individuals, rarely used illicit non-opioid substances. However, the absence of these two features does not explain the results obtained: the reason is that in China, there was little or no availability of illicit non-opioid substances in the 1990s, and that in both China and Germany, accessibility to methadone treatment was low or absent during this period. In any case, the negative results reported by [19] could also be related with the low severity of the SUD assessed, because this research group examined associations with opioid abuse, rather than opioid dependence, as did the remaining groups.

In the other previous study in which genetic associations were detected, all participants were males [18]. As this study did, we found allelic and genotypic associations in our male subsample. However, the female participation in our study was useful because it indicated that the genotypic association was not gender related, whereas the allelic association was indeed male-limited. The latter association could be related to the finding that nonspecific genetic factors account for much more variance percentage of illicit opioid use in males (37%) [4] than in females (3%)[3].

Moreover, gender comparisons in the present study showed that cannabis use was more frequent in males than in female patients, and the same trend was found regarding alcohol use. These differences suggest that our decision to include patients who use non-opioid substances in the study maximised the odds for detecting genetic associations more in males than in females. However, associations between the *DRD2 *A1 allele and cannabis use disorders have not been published. Moreover, nonspecific genetic factors account for a larger fraction of the variance of cannabis use in females (59%)[3] than in males (34%) [4]. Male-limited associations of the *DRD2 *A1 allele with alcohol dependence [26] and tobacco smoking [27] have been reported previously.

Finally, the present study has the following limitations:

1) The associations found could be related more to the progression and severity of heroin dependence than vulnerability to this disorder [28], because patients had a long history of heroin use; 2) Type II error is a potential explanation for our findings, since the differences detected did not reach the significance threshold (i.e.: *P *< 0.008) when the Bonferroni correction was applied to the six genetic comparisons performed (Tables 2 and 3); however, type II error could occur in any direction (i.e., either A1 or A2 could have been associated with heroin use), while we found that the associated allele was A1, just as in previous reports; 3) The male-limited association of A1 allele with heroin dependence must be considered as a preliminary finding, because more males than females participated in the present study and there is no indication of gender dimorphism in regard with the *Taq*I A alleles in the literature; 4) The findings of the present study may not be generalizable, because all study participants belonged to the same population. Still, Spaniards are closely related to the other European populations, and one would not expect to find many differences in the genetic architecture of susceptibility to heroin use.

## Conclusion

To our knowledge, this is the first study performed in Latin Europe regarding the genetic associations of heroin dependence. The study was aimed to examine the association of heroin dependence with the *DRD2 Taq*I A polymorphism in Spanish subjects of European origin. According with our results, the A1-A1 genotype of the *DRD2 Taq*I A polymorphism is associated with heroin dependence regardless of gender, while the A1 allele is associated with heroin dependence only in males. These results must be considered taking into account two aspects of the participant selection. First, heroin-dependent patients who participated in the present study were not excluded if they reported non-opioid substance use. Second, the most of cases and controls were males.

## Abbreviations

*DRD2*: dopamine D2 receptor gene

SUD: substance use disorders

## Competing interests

The author(s) declare that they have no competing interests.

## Authors' contributions

JP made substantial contributions to the conception and design of the study, the interpretation of data, and drafting of the manuscript. MB made substantial contributions to the conception and design of the study, oversaw genotyping, and helped to draft the manuscript.

JT made substantial contributions to the conception and design of the study, assisted with the analysis and interpretation of the statistical data, and revised the manuscript. NS performed the statistical analyses, contributed to the interpretation of the statistical data, and revised the manuscript. VV and FC assisted with statistical analyses, contributed to the interpretation of the statistical data, and revised the manuscript. EB and EL participated in recruiting part of subjects and phenotyping of subjects. ER performed the genotyping analyses. EA made substantial contributions to the conception of the study, and helped to draft the manuscript. All authors read and approved the final manuscript.

## References

[B1] Tsuang MT, Lyons MJ, Meyer JM, Doyle T, Eisen SA, Goldberg J, True W, Lin N, Toomey R, Eaves L (1998). Co-occurrence of abuse of different drugs in men: the role of drug-specific and shared vulnerabilities. Arch Gen Psychiatry.

[B2] Merikangas KR, Stolar M, Stevens DE, Goulet J, Preisig MA, Fenton B, Zhang H, O'Malley SS, Rounsaville BJ (1998). Familial transmission of substance use disorders. Arch Gen Psychiatry.

[B3] Karkowski LM, Prescott CA, Kendler KS (2000). Multivariate assessment of factors influencing illicit substance use in twins from female-female pairs. Am J Med Genet.

[B4] Kendler KS, Jacobson KC, Prescott CA, Neale MC (2003). Specificity of genetic and environmental risk factors for use and abuse/dependence of cannabis, cocaine, hallucinogens, sedatives, stimulants, and opiates in male twins. Am J Psychiatry.

[B5] Wise RA, Bozarth MA (1987). A psychomotor stimulant theory of addiction. Psychol Rev.

[B6] Koob GF, Le Moal M (1997). Drug abuse: hedonic homeostatic dysregulation. Science.

[B7] Maldonado R, Saiardi A, Valverde O, Samad TA, Roques BP, Borrelli E (1997). Absence of opiate rewarding effects in mice lacking dopamine D2 receptors. Nature.

[B8] Noble EP, Blum K, Ritchie T, Montgomery A, Sheridan PJ (1991). Allelic association of the D2 dopamine receptor gene with receptor-binding characteristics in alcoholism. Arch Gen Psychiatry.

[B9] Thompson J, Thomas N, Singleton A, Piggott M, Lloyd S, Perry EK, Morris CM, Perry RH, Ferrier IN, Court JA (1997). D2 dopamine receptor gene (DRD2) Taq1 A polymorphism: reduced dopamine D2 receptor binding in the human striatum associated with the A1 allele. Pharmacogenetics.

[B10] Pohjalainen T, Rinne JO, Nagren K, Lehikoinen P, Anttila K, Syvalahti EK, Hietala J (1998). The A1 allele of the human D2 dopamine receptor gene predicts low D2 receptor availability in healthy volunteers. Mol Psychiatry.

[B11] Laruelle M, Gelernter J, Innis RB (1998). D2 receptors binding potential is not affected by Taq1 polymorphism at the D2 receptor gene. Mol Psychiatry.

[B12] Blum K, Sheridan PJ, Wood RC, Braverman ER, Chen TJ, Cull JG, Comings DE (1996). The D2 dopamine receptor gene as a determinant of reward deficiency syndrome. J R Soc Med.

[B13] Volkow ND, Wang GJ, Fowler JS, Logan J, Gatley SJ, Gifford A, Hitzemann R, Ding YS, Pappas N (1999). Prediction of reinforcing responses to psychostimulants in humans by brain dopamine D2 receptor levels. Am J Psychiatry.

[B14] Kaasinen V, Nagren K, Hietala J, Farde L, Rinne JO (2001). Sex differences in extrastriatal dopamine D_2_-like receptors in the human brain. Am J Psychiatry.

[B15] Wang E, Ding YC, Flodman P, Kidd JR, Kidd KK, Grady DL, Ryder OA, Spence MA, Swanson JM, Moyzis RK (2004). The genetic architecture of selection at the human dopamine receptor D4 (DRD4) gene locus. Am J Hum Genet.

[B16] Young RM, Lawford BR, Nutting A, Noble EP (2004). Advances in molecular genetics and the prevention and treatment of substance misuse: Implications of association studies of the A1 allele of the D2 dopamine receptor gene. Addict Behav.

[B17] Lawford BR, Young RM, Noble EP, Sargent J, Rowell J, Shadforth S, Zhang X, Ritchie T (2000). The D2 dopamine receptor A1 allele and opioid dependence: association with heroin use and response to methadone treatment. Am J Med Genet.

[B18] Shahmoradgoli Najafabadi M, Ohadi M, Joghataie MT, Valaie F, Riazalhosseini Y, Mostafavi H, Mohammadbeigi F, Najmabadi H (2005). Association between the DRD2 A1 allele and opium addiction in the Iranian population. Am J Med Genet B Neuropsychiatr Genet.

[B19] Li T, Liu X, Zhao J, Hu X, Ball DM, Loh el-W, Sham PC, Collier DA (2002). Allelic association analysis of the dopamine D2, D3, 5-HT2A, and GABA_A_γ2 receptors and serotonin transporter genes with heroin abuse in Chinese subjects. Am J Med Genet.

[B20] Xu K, Lichtermann D, Lipsky RH, Franke P, Liu X, Hu Y, Cao L, Schwab SG, Wildenauer DB, Bau CH, Ferro E, Astor W, Finch T, Terry J, Taubman J, Maier W, Goldman D (2004). Association of specific haplotypes of D2 dopamine receptor gene with vulnerability to heroin dependence in 2 distinct populations. Arch Gen Psychiatry.

[B21] American Psychiatric Association (1994). Diagnostic and Statistical Manual of Mental Disorders.

[B22] Plan Nacional sobre Drogas (PND) (2002). Observatorio Español sobre Drogas. Informe n° 5 – Julio.

[B23] Miller SA, Dykes DD, Polesky HF (1988). A simple salting out procedure for extracting DNA from human nucleated cells. Nucleic Acids Res.

[B24] Grandy DK, Zhang Y, Civelli O (1993). PCR detection of the TaqA RFLP at the DRD2 locus. Hum Mol Genet.

[B25] Caplehorn JRM, Saunders JB (1993). A comparison of residential heroin detoxification patients and methadone maintenance patients. Drug Alcohol Rev.

[B26] Limosin F, Gorwood P, Loze JY, Dubertret C, Gouya L, Deybach JC, Ades J (2002). Male limited association of the dopamine receptor D2 gene TaqI A polymorphism and alcohol dependence. Am J Med Genet.

[B27] Lee HS (2003). Gender-specific molecular heterosis and association studies: dopamine D2 receptor gene and smoking. Am J Med Genet B Neuropsychiatr Genet.

[B28] Daly AK (2003). Candidate gene case-control studies. Pharmacogenomics.

